# Intrinsic dimensionality of human behavioral activity data

**DOI:** 10.1371/journal.pone.0218966

**Published:** 2019-06-27

**Authors:** Luana Fragoso, Tuhin Paul, Flaviu Vadan, Kevin G. Stanley, Scott Bell, Nathaniel D. Osgood

**Affiliations:** 1 Department of Computer Science, University of Saskatchewan, Saskatoon, Saskatchewan, Canada; 2 Department of Geography and Planning, University of Saskatchewan, Saskatoon, Saskatchewan, Canada; 3 Department of Community Health and Epidemiology, University of Saskatchewan, Saskatoon, Saskatchewan, Canada; University of Warwick, UNITED KINGDOM

## Abstract

Patterns of spatial behavior dictate how we use our infrastructure, encounter other people, or are exposed to services and opportunities. Understanding these patterns through the analysis of data commonly available through commodity smartphones has become an important arena for innovation in both academia and industry. The resulting datasets can quickly become massive, indicating the need for concise understanding of the scope of the data collected. Some data is obviously correlated (for example GPS location and which WiFi routers are seen). Codifying the extent of these correlations could identify potential new models, provide guidance on the amount of data to collect, and even provide actionable features. However, identifying correlations, or even the extent of correlation, is difficult because the form of the correlation must be specified. Fractal-based intrinsic dimensionality directly calculates the minimum number of dimensions required to represent a dataset. We provide an intrinsic dimensionality analysis of four smartphone datasets over seven input dimensions, and empirically demonstrate an intrinsic dimension of approximately two.

## Introduction

How people move through and make use of space is fundamental to disciplines as disparate as Architecture, Public Health, Civil Engineering and City Planning [1], and informs outcomes from the spread of contagious disease to the role of transit in moving people from place to place [2, 3]. Models of how space is accessed and used have traditionally been derived from survey [4] or observational data [5], but in the last 20 years, electronically mediated data acquisition has become the norm [6, 7], enabled by advances in GPS positioning [8], indoor positioning [9] and distributed sensing devices through body sensor networks and the Internet of Things [10, 11].

The mass adoption of the smartphone over the last decade has opened new research horizons for researchers interested in studying spatial behaviour, given the diverse array of sensors available on the phone and the ability of the phone to issue short surveys [6], often known as ecological momentary assessments. The radios from the GPS, cellular and WiFi sensors can be leveraged through trilateration or related techniques to provide location [12, 13]; accelerometers, gyroscopes and magnetometers can be used to infer types of activity [14, 15] and modes of transportation [16]. Screen state, camera and app usage data can support inference of aspects of a person’s affect [17] and personality [18]. Even the charging behaviour can be linked with important metrics, such as those related to sleep patterns [19].

The change in data volume and quality has changed the way that analysis must unfold. While traditional survey and observational-based methods could be treated with frequency-based statistical tests [4, 5] and simple analysis of location traces gathered through technologies such as GPS can be manipulated using spatial statistics [12], diverse datasets employing data from a number of high velocity sensor can be more difficult to analyze at scale [20]. A myriad of statistical and machine learning techniques have been used to map smartphone-derived sensor data to actionable outcomes [1], but most make the assumption that each channel offers incremental information beyond the last. While some measures are almost certainly correlated (for example, GPS location and the visibility of specific cellular towers or WiFi hotspots), occurrence of other co-variations are less obvious, and likely to be more textured (for example, app use and activity level). As described by Camastra [21], the use of unnecessary dimensions can result in many problems such as space to store the data, speed performance of algorithms, and building good classifiers due to the curse of dimensionality.

Some initial studies on the incremental information value of different spatio-temporally correlated measures have been contributed. In particular, linear methods such as principal component analysis (PCA) have been employed to determine which dimensions of behaviour account for most of the variance within different measured dimensions of spatial behaviour [14]. However, these methods do not paint an accurate picture of the fundamental correlation between dimensions as they are limited by their underlying assumptions, often linearity, normality or stationarity [22, 23]. Neural networks are a common alternative approach for non-linear PCA but also showed performance limits [24]. On the other hand, applying PCA locally in non-linear manifolds can be found in algorithms such as local-PCA [25] and OTPMs PCA [26]. However, these algorithms do not guarantee to cover the whole dataset. Isomap [27] and C-PCA [23] algorithms, based on some PCA features and the nearest neighbor (NN) distances, do guarantee a global estimation by preserving the distances of the original data. While these methods provide algorithmically actionable measures of dimensionality, they explicitly do not provide insight into the minimum possible number of dimensions required to represent a function, stochastic variable or dataset [21, 28], also called intrinsic dimensionality (ID).

More reliable ID global estimators have been proposed. Costa and Hero [29] extended the ISOMAP algorithm by creating a graph of the data sample and then pruning it to the minimal spanning graph (GMST). The ID is estimated based on the GMST length. Maximum likelihood estimator [28] is another global estimator based on probabilistic assumptions. Among these ID estimators, fractal-based methods are extensively explored in the literature and have been shown to be a good ID estimators [30, 31]. As shown by Lee and Verleysen [31], among PCA, local-PCA, “trial and error” method, and a fractal-based method, the latter clearly resulted in the best estimation of ID. They also have a well-established record of utility outside of GIS and has been used for to inform both state space and time series models of behaviour [32], particularly in the health science [33]. The Box Counting dimension is one of the most popular fractal-based methods [21]. Camastra and Vinciarelli [30] proposed a fractal-based method to estimate the ID of a dataset using the correlation dimension definition [34] as a substitute for the Box Counting dimension due to its computational simplicity. On the other hand, Traina Jr. et al. [35] addressed the Box Counting dimension by developing a multi-level grid structure. Each cell of the grid is represented by the number of data points that are within the location of the cell, and if a cell has at least one data point, a new grid is generated for the next level with the cells containing half of the size. Their algorithm showed a linear computational cost in relation to the number of dataset points (*N*), that is, O(*N*). However, storage cost still remained a problem with a complexity of O(*N*) and Wong et al. [36] proposed a fractal-based algorithm, Tug-of-War, with the same computational cost, O(*N*), but with a storage cost of O(1).

When narrowing the use of ID for human behavior, previous studies have usually focused on visual tracking, using body-sensors or cameras [37–39]. There is a lack of studies applying intrinsic dimensionality for smartphone sensors to understand human behavioral activity more broadly. Investigating ID of datasets is the province of complexity theory [23]. Using a single metric for analyzing human behavior and spatial complexity of data is desirable as it can describe the overall complexity of a data set and inform model design, data collection and interpretation. For instance, large dimensionalities indicate low predictability, and low likelihood of model building success. Lower dimensional spaces are more likely to be amenable to modelling and analysis. ID for human behavior can also be used as a metric to represent relative information content between populations. Populations which exhibit little covariance across measured dimensions are represented by larger dimensions and conversely, populations which exhibit strong correlation between measured features exhibit lower dimensionality.

In this paper, we establish the viability of the approach of analyzing smartphone mobility and activity data using dimensionality and provide baseline insight into its value and form over seven features and four datasets. To calculate their ID, we apply a similar Box Counting dimension implementation to the one described by Traina Jr., Traina, and Wu [35] but, unlike Tug-of-War [36], we do not improve the storage cost, because our datasets are not large enough to encounter memory issues on modern computers. Using a tree decomposition, we are able to probe the structure of the dimensionality, a novel step in dimensionality analysis of datasets. We demonstrate that for the datasets under consideration between 1.82 and 1.90 dimensions are fundamentally required to represent the data. Post-hoc PCA analysis leads to some additional insight, but is inconclusive on the structure of the dimensions, indicating that the relationships between the human behavioral activity dimensions in the limit are non-linear. With the post-hoc PCA analysis, we show that researches need to exercise care when assuming that human behavioral data is linearly correlated, and a non-linear method is preferable but do not propose such a method because Box Counting provides an estimate of the ID, but not the parameters which contribute to that minimum representation of the data. We use PCA to contrast the difference between the number of dimensions computed under linear and non-linear assumptions for the data in question.

## Experimental setup

Our investigation sought to determine the intrinsic dimensionality (ID) of four different datasets containing smartphone sensor metrics using the Box Counting dimension (this algorithm is explained under the section *Intrinsic Dimensionality*). We selected 7 dimensions that were presented across the four datasets and described human behavioral activity: time (only hour), latitude, longitude, acceleration, standard deviation of acceleration, battery status, and WiFi connectivity. We aggregated, merged, filtered, and normalized the datasets. The final number of records of each dataset varied from 109,986 to 231,382. We then inserted these records in a dataset-specific n-D Tree structure to calculate the required parameters for the Box Counting dimension. The slope of the log-log plot of these data points was calculated to estimate the ID as proscribed in [21, 35, 40].

### Datasets

Several human behavioral studies [3, 6, 7, 9] have used the Saskatchewan Human Ethology Datasets (SHEDs) that contain various smartphone-sourced sensor records. In this paper, we used the four most recent SHEDs: 7, 8, 9, and 10, which were collected via the Ethica mobile app [41]. The study was approved by the University of Saskatchewan Behavioural Ethics Board, with reference to file number BEH 14-293. The form of consent was written. Each participant had a unique code assigned to his/her name for the sake of anonymity and the data were structured in MySQL (version 14.14, distribution 5.5.24) database. The collection duration and count of participants and records for each SHED is provided in Table 1.

**Table 1:** Datasets information.

	SHED7	SHED8	SHED9	SHED10
Duration (days)	38	30	40	29
Participants	63	75	87	108
Records *	496M	450M	2.8B	600M

* M = million, B = billion

Ethica [41] collects sensor data using a duty cycle, typically for one minute every five, over a number of smartphone measures including but not limited to location, activity and phone use. Additional datastreams such as phone orientation, activity type, and GIS measures such as convex hull are either directly available or computable through post-processing. Because this work is meant to establish the viability of the Box Counting approach and to study its utility, we selected seven relatively low level features available across all datasets. Because geographers have long established the importance of time and place [42, 43], we selected hour of the day, latitude and longitude as key indicators. We additionally report the number of WiFi nodes visible in a duty cycle. While this is not directly a measure of location, it is likely to correlate with location, as participants are likely to see the same WiFi routers in the locations which they regularly visit. Phones are also often used to measure activity [1, 14, 15]. Raw measurements from the accelerometer and gyroscope are seldom useful for direct conclusions about activity, but the mean and variance of the norm of the acceleration has been shown to loosely correlate with whether or not a participant is physically active. Finally, we record the battery state (charging or discharging). This will loosely correspond to both time and location (people tend to plug phones in at the same time and place, for example, at bedside before sleeping), as well as phone use (greater use leading to faster depletion and more frequent charging) [19]. Between these columns we have captured, at least tangentially, the three most common uses of smartphone telemetry: mapping people through time and space, recording and reporting on activity, and tracking use of the phone itself. On the surface, these would appear to be distinct classes of measurement, only tangentially related, which can be appropriately probed with a dimensionality analysis, as opposed to a set of parameters that were clearly correlated, or impossible to correlate. Latitude and longitude are measurements of continuous variables, at least to the noise floor of the sensor, as are the norm and variance of the accelerometer, further indicating the potential utility of ID as a measure of dataset complexity.

#### Data preprocessing

We chose to use the the five-minute Ethica duty cycle noted above as the time quantum for analysis. However, most data streams report multiple values over the minute that the duty cycle is active. We therefore aggregated each data stream for each participant to the duty cycle level for each metric, such that for each duty cycle there was at most a single entry for a participant. By aggregating, we achieved a consistent number of records per duty cycle and summarized the information. Consequently, the noise in signals, such as GPS, were suppressed which is likely beneficial as we are interested in analyzing human mobility, not GPS precision. In contrast to the other data sources, the GPS table is not guaranteed to contain an entry for every participant duty cycle, because the participant could have been indoors and unable to receive a GPS satellite signal. In cases where no GPS data was reported, the entire record was ignored, biasing our analysis towards outdoor behavior. Data was then merged across participants and duty cycles such that an entire dataset with all seven features was contained in a single table. The resulting datasets were filtered and normalized. Normalization bounds were determined within a dataset, but across participants. Python 3.6 with pandas 0.20.3 was used for the filtering process, and Java 1.8 for the aggregation and normalization. The criteria for aggregation, filtering, and normalization (feature scaling) for each of the 7 features are described below:

**Timestamp** For aggregation, we took the most recent timestamp and mapped it to the hour of the day, which was normalized to the range *hour* ∈ [0, 1].
**Longitude and Latitude** We took the last record (the most recent record) when aggregating to the duty cycle level. Due to limitations of GPS localization caused by nominal array of satellite connectivity [3], we decided to filter our data to the bounds of Saskatoon, Saskatchewan, Canada, where the SHED datasets were predominantly collected. Therefore, any longitude values less than -106.7649138128 and greater than -106.52225318, and any latitude values less than 52.058367 and greater than 52.214608, were removed. After the filtering, the values of *lon* and *lat* were normalized, *lon* and *lat* ∈ [0, 1].
**Acceleration Norm** Since this metric is composed of acceleration in the *x, y* and *z* directions with respect to the phone, acceleration was averaged over each duty cycle and combined across spatial dimensions using the L2 norm. Outliers greater than three standard deviations from the mean were removed. The acceleration norm was then normalized between 0 and 1 for each dataset.
**Standard deviation of acceleration** The standard deviation of the acceleration over a time window can be used as a simple measure of whether a person is active. We calculated the L2 norm for each accelerometer reading during each timestep as above, and then calculated their standard deviation over a single duty cycle. Outliers greater than three standard deviations from the mean were removed. Acceleration standard deviation was then normalized between 0 and 1 for each dataset.
**Battery Status** The most recent record was considered when aggregating the data. The battery status records whether a mobile device is “Charging AC”, “Charging USB”, “Charging Wireless”, or “Not Charging”, and is a useful proxy for whether the phone is being carried by the participant. With the normalization, 0 was assigned to “Not charging”, 0.25 to “Charging AC”, 0.5 to “Charging USB”, and 1 for “Charging Wireless”.
**WiFi connectivity** For aggregation, we counted the number of unique WiFi router MAC addresses (*wifi*) that a participant observed in a given duty cycle. Any entry that had a *wifi* count of 0 was removed. Then, the value of *wifi* was normalized, *wifi* ∈ [0, 1].

The final step was to remove duplicates, since there were rare cases where two or more data points presented the same values for the 7 selected dimensions, which can confuse the Box Counting algorithm. Table 2 shows the number of participants and records for each dataset before and after selecting the sensor metrics of interest, filtering and normalizing the data.

**Table 2:** Dataset information before and after data preprocessing.

		Before	After
SHED7	Participants	63	63
	Records *	496M	170,754
SHED8	Participants	75	60
	Records	450M	109,986
SHED9	Participants	87	86
	Records	2.8B	231,382
SHED10	Participants	108	105
	Records	600M	135,814

* M = million, B = billion

### Intrinsic dimensionality

Intrinsic dimensionality (ID) is the minimum number of dimensions required to represent a dataset without losing information [30]. Based on the definition provided by [25, 30], an ID of a dataset with *d* nominal dimensions is equal to *M* only if all of the dataset elements lie within a minimal *M*-dimensional subspace of *d* (where 0 ≤ *M* ≤ *d*).

A popular approach to obtain ID is the Box Counting dimension, a fractal-based method, which is a simplified version of the Hausdorff dimension [21]. For the rest of the document, we refer to Box Counting dimension as dimensionality for brevity. To calculate the dimensionality of a set *F*, we draw a number of hypercubes of side length *ε* and count the number of hypercubes, *N*(*ε*), that cover the set *F* for decreasing values of *ε*. The dimensionality is defined as follows:

$$\begin{array}{c} D_{box}=\lim_{\varepsilon \to 0}\frac{\log N(\varepsilon )}{\log\frac{1}{\varepsilon }} \end{array}$$ (1)

where *ε* represents the side length of the hypercube and *N*(*ε*) is the number of hypercubes of side length *ε* containing data. Since the dimensionality is applied for different sizes of *ε*, ID is estimated based on how *N*(*ε*) changes as *ε* becomes finer. In addition, as pointed by Camastra [21], it has been proven that the following should be true to obtain an accurate ID estimation:

$$\begin{array}{c} d<2\log_{10}\;n \end{array}$$ (2)

where *d* is the nominal number of dimensions and *n* is the number of records. The inequality 2 is satisfied for all of the datasets in consideration in our work, SHED7-10, with *d* = 7 and *n* as shown in Table 2, where 2 log_10_ *n* is always greater than 10.

Based on Eq 1, the data needs to be structured in hypercubes for different *ε* values. Therefore, we developed an n-Dimensional (n-D) Tree, which allowed us to regularly specify a hypercube in more concrete terms for different values of *ε*. The following subsections explain in details the n-D Tree decomposition and how we estimated the ID. Our algorithm is presented in Algorithm 1, which was developed in Python 3.6.1 with the packages pandas 0.20.3 and numpy 1.13.1.

**Algorithm 1** Dimensionality implementation

**input**: normalized dataset *S* with *d* dimensions

**output**: intrinsic dimensionality *M*

1: **for each** tuple *t* = (*r*_1_, *r*_2_, …, *r*_*d*_) in *S* **do**

2:  **while** hypercube H with side length *ε* is not a leaf **do**

3:   Insert *t* into *H*

4:   Find any appropriate hypercube child *Hc* into whose range *t* falls

5:   **if** *Hc* exists **then**

6:    *H* = *Hc*

7:   **end if**

8:  **end while**

9:  **if** *H* has no data **then**

10:   Insert *t* into *H*

11:  **else**

12:   Split *H* into (2^*d*^) *Hc* each of side length $\frac{\varepsilon }{2}$

13:   Insert the points of *H* into the appropriate *Hc*

14:   Insert *t* into *H* and the appropriate *Hc*

15:   **if** *Hc* has more than one data **then**

16:    *H* = *Hc*

17:    Repeat steps 12 to 14

18:   **end if**

19:  **end if**

20: **end for**

21: Count *N*(*ε*) for each *ε*

22: Fit a line in the linear part of the log-log plot of *N*(*ε*) vs. $\frac{1}{\varepsilon }$

23: *M* = slope of this line

24: Return *M*

#### n-D Tree decomposition

Similar to Traina Jr. et al. [35], we represented a *d* dimensional hypercube as a node of a tree structure that we termed an n-Dimensional (n-D) Tree, which allowed the insertion of participant records (data points) according to the coordinate ranges of the hypercubes to which they belonged. However, while Traina Jr. et al. [35] assumes that the hypercubes’ positions are always known, we calculated their positions for each *ε*. In their methodology, each hypercube only informs the sum of the data points it possesses, in contrast, we chose to insert the data points into the hypercubes so that we have the structure of our data. Therefore, our participant’s records and hypercubes’ coordinates had the general form (*r*_1_, *r*_2_, …, *r*_*d*_) and [(*a*_1_, *b*_1_), …, (*a*_*d*_, *b*_*d*_)], respectively, where *d* is the maximum number of dimensions, in our case 7, *r*_*d*_ is the participant record at dimension *d*, and *a*_*d*_ and *b*_*d*_ are the minimum and maximum values of the coordinates for the respective dimension *d*. A data point belongs to a hypercube if it is greater than *a*_*d*_ and less than or equal to *b*_*d*_, which allowed the special case of having a data point on one of the hypercube’s axis. For the sake of efficiency, we constrained each coordinate axis to the range of [0, 1], motivating normalization of our data.

**Fig 1:**
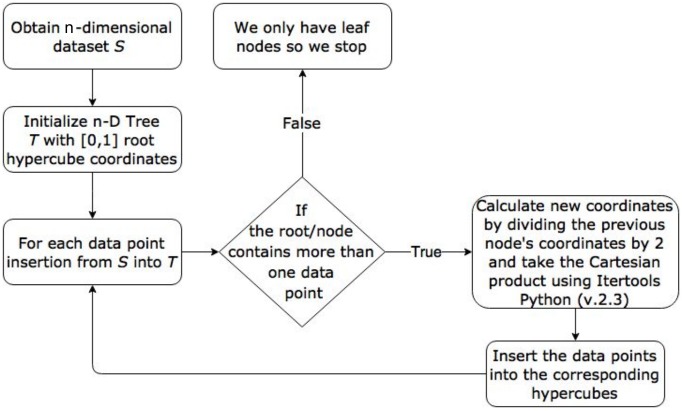
n-D Tree data insertion algorithm.

All data points are encompassed by the root node as it spans [0, 1] on all dimensions. The root node of the n-D Tree represented the spanning hypercube, which was then subdivided into smaller hypercubes. The condition of containing more than one data point splits the hypercube into 2^*d*^ hypercube children with the half of the *ε* size. Therefore, a new set of range coordinates had to be calculated for each child, based on the previous hypercube’s coordinates. For every axis representing a range, we used the lower bound and the upper bound to calculate the new coordinates. The lower and upper bounds were added and divided by 2 to obtain the middle point. As a result, new coordinates were a tuple set of the form [(lower, middle), (middle, top)]. Once we created a list of all the new possible coordinates, we used the Python package Itertools (version 2.3) to return the Cartesian product of the new coordinates. Finally, the inserted points on the previous hypercube were re-inserted into the newly allocated and corresponding hypercube children. This method of continuously partitioning the initial hypercube allowed us to keep the originally inserted data points in the leaves since they are the nodes containing only one data point. It is worth noting that this method has a larger memory footprint than other methods, but provides the benefit of post-hoc analysis of the tree structure. Fig 1 presents a flowchart of our n-D Tree method.

With the n-D Tree, it was possible to calculate the parameters in Eq 1. The proportion containing data is critical for calculating the *N*(*ε*) parameter, reflecting the fact that

$$\begin{array}{c} \varepsilon =\frac{1}{d(2^{l})} \end{array}$$ (3)

where *l* is the current level and *d* is the nominal dimension of the dataset. Since our algorithm does not expand nodes with only one data point (leaf), this data is not re-inserted in the next levels. Therefore, *N*(*ε*) was calculated by summing the number of nodes with data at the corresponding level plus the number of previous leaves, so that all the data points were considered for each *ε*.

#### Determining ID

From different proposed methods, the most widely used one in the literature to estimate the Box Counting is the slope of the linear part of *log*(*N*(*ε*)) vs. *log*(1/*ε*) [21, 35, 40]. To select the linear part of the log-log plot, we found the level of the tree where the number of nodes containing data begins to decrease, because after this level, *N*(*ε*) increases slowly as *ε* decreases, resulting in an asymptotic rather than linear curve. Because the asymptotic curve starts between the points where the tree switches from increasing to decreasing number of nodes with data, we fitted a line for both cases and took the average of their slopes as the final ID value. We used the numpy (version 1.13.1) function polyfit to fit the lines.

## Results

We calculated the intrinsic dimensionality over the four datasets: SHED7, SHED8, SHED9, and SHED10 on a MacBook Pro 2.7 GHz Intel Core i7 with 16 GB 1600 MHz DDR3 RAM. Table 3 shows the runtime and memory usage of our algorithm for each dataset, not including data download time from the database. Run time accounts for the building of the tree and calculation of the parameters in Eq (1). We analyzed the n-D Tree structure value and, finally, analyzed the PCA results under a linear assumption to contrast with the Box-counting algorithm. Figures were plotted using Python matplotlib 2.0.2.

**Table 3:** Performance report.

	SHED7	SHED8	SHED9	SHED10
Runtime (min)	13	10	25	12
Memory usage (GB)	7	5	10	6

### n-D Tree structure

**Fig 2:**
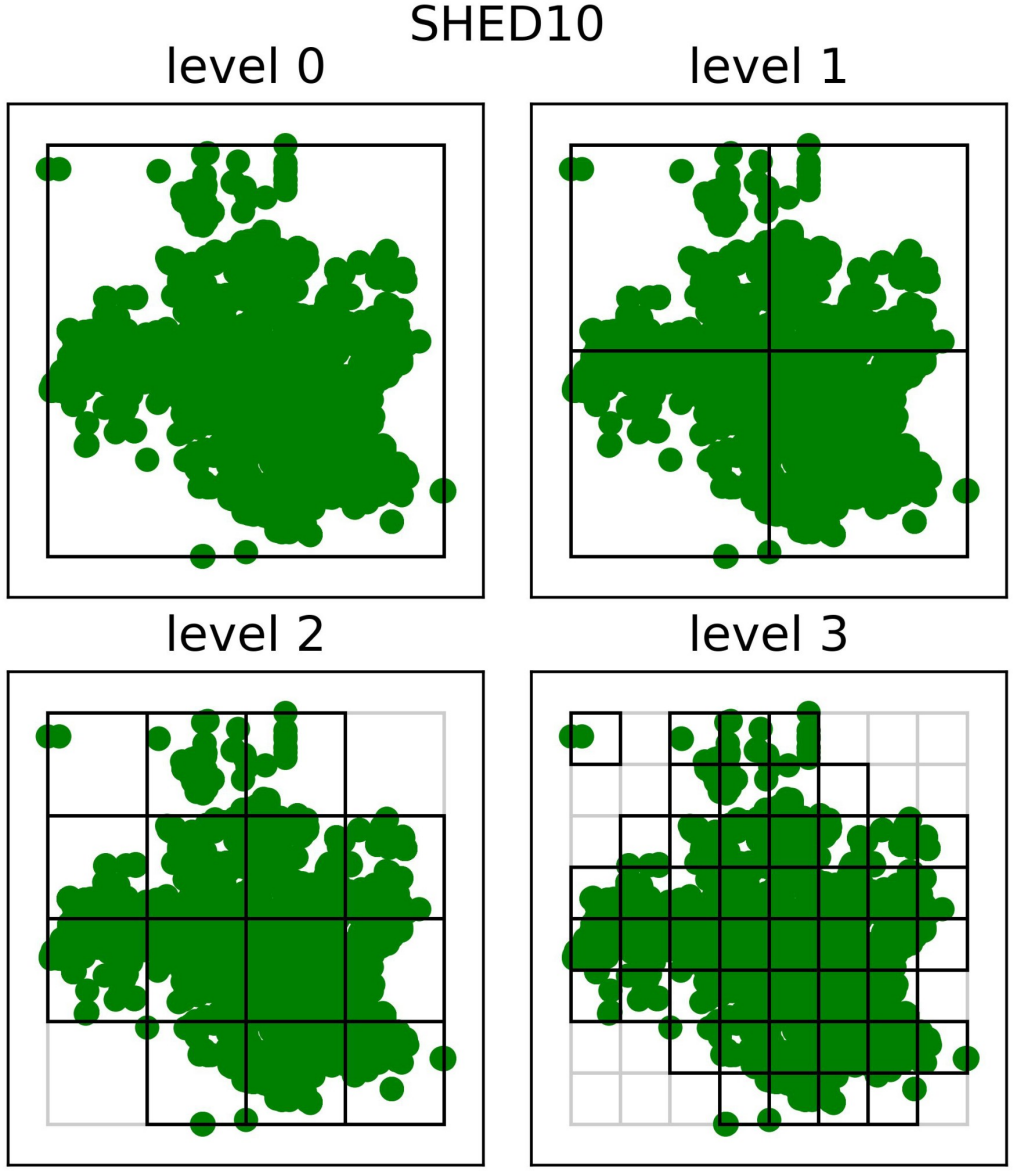
Quad-Tree of SHED10. Latitude and longitude partitioned in the first four levels.

Analyzing n-D Tree sparsity is important to identify how the data points are concentrated and, therefore, how the dataset can be better described. As a result, we first analyzed how our 7-D Tree partitioned the data points for each dataset. Fig 2 shows how the latitudes and longitudes were partitioned for the first four levels of SHED10, the most recent one of the SHEDs table. Each cell represents a node of the tree, and the ones in bold are the nodes containing data in the respective level. We can see that the tree becomes sparser as it becomes deeper. All datasets exhibit the following trend: in levels 0 and 1, 100% of the nodes contain data, while in levels 2 and 3, this is not true.

**Fig 3:**
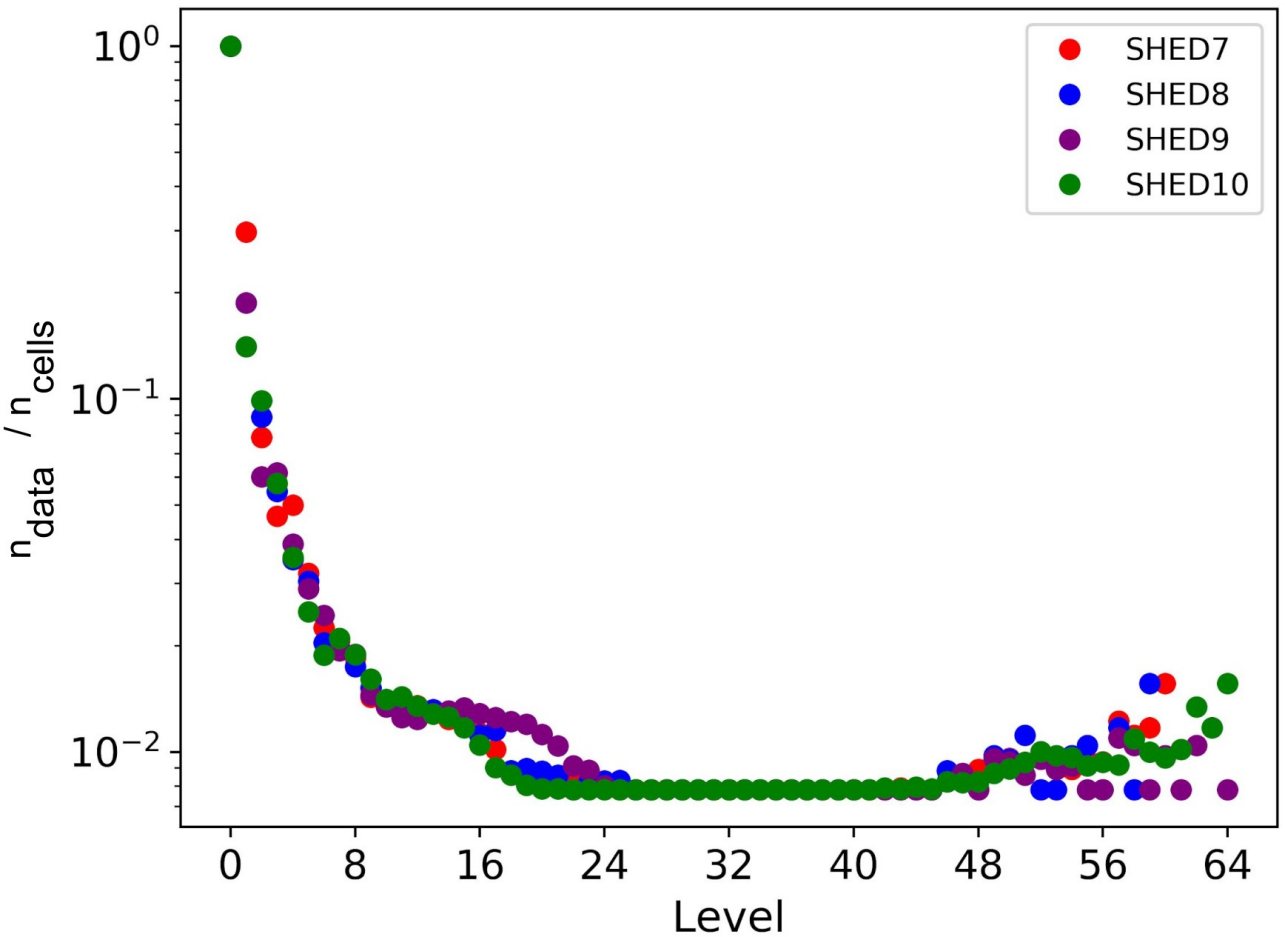
Proportion of nodes with data per level.

We then further investigated this trend for all the 7 dimensions by calculating the proportion of nodes containing data (*n*_*data*_) in relation to the total number of nodes (*n*_*cells*_) in each level. As shown in Fig 3, all of the original data points were inserted into the leaves with no more than 64 levels, *ε* = 7.74E-21. The proportion of nodes with data decays abruptly until the 8^th^ level (*ε* = 0.0005), indicating that the rate of insertion is declining. Some of these data were similar because at least two data points were inserted in the same hypercube between the 24^th^ and 48^th^ levels (*ε* = 8.51E-09 and *ε* = 5.07E-16, respectively). Only after the 48^th^ level, was the range sufficiently fine to separate these data points into different hypercubes, showing an increase of the proportion of nodes with data. We believe that these similar data were the result of GPS and accelerometer noise. Because dimensionality is only valid for data above the noise floor, we ignore tree levels greater than 24 in subsequent trend analysis. The trend in Fig 3 can be described as $\frac{n_{data}}{n_{cells}}=c^{level}$, where 0.14 < *c* < 0.3, according to Eureqa [44], with a *R*^2^ goodness of fit around 0.99. However, we note that the *R*^2^ value must be regarded with caution as the involved equations are not linear.

**Fig 4:**
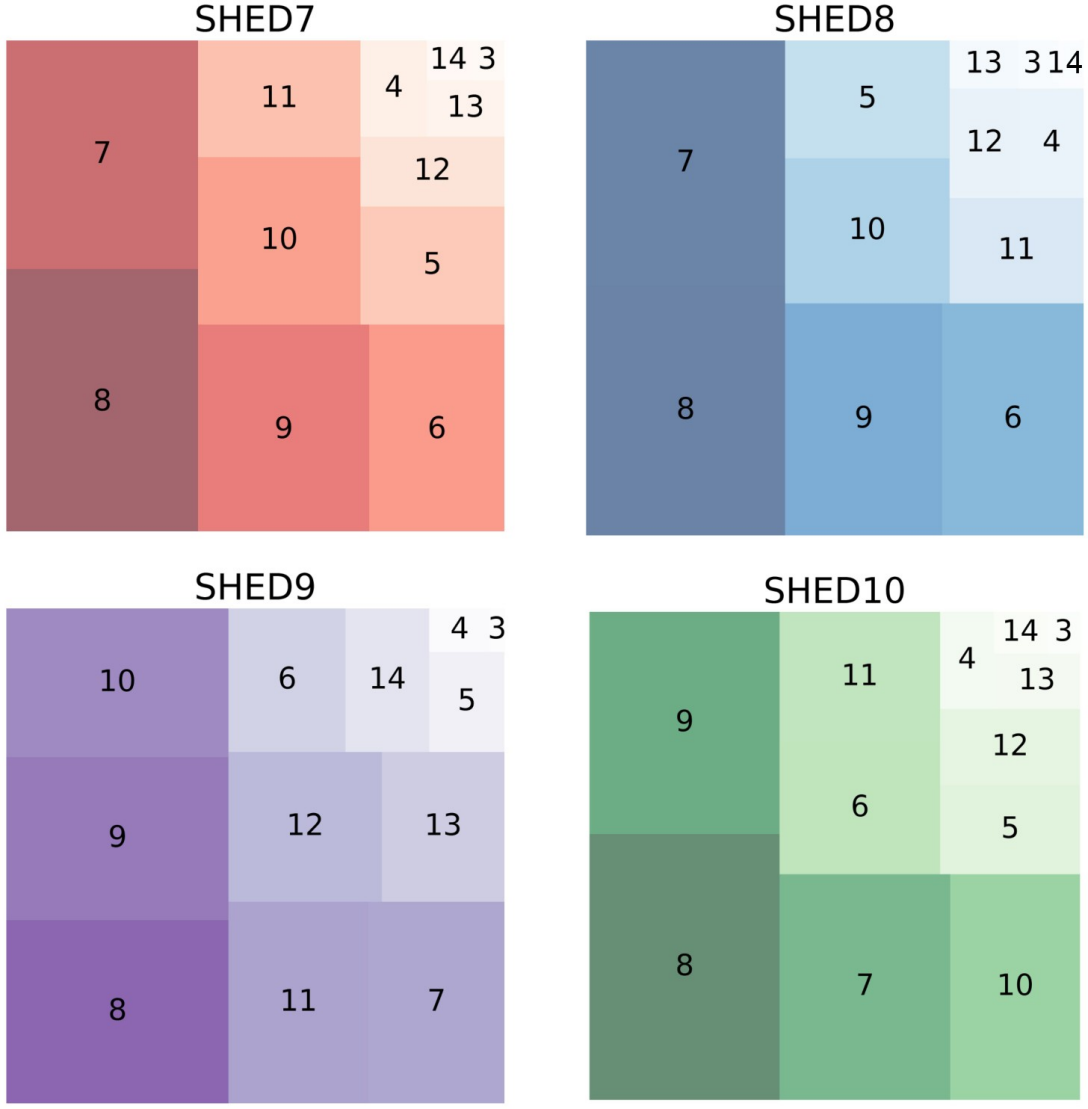
TreeMap of nodes with data per level.

**Fig 5:**
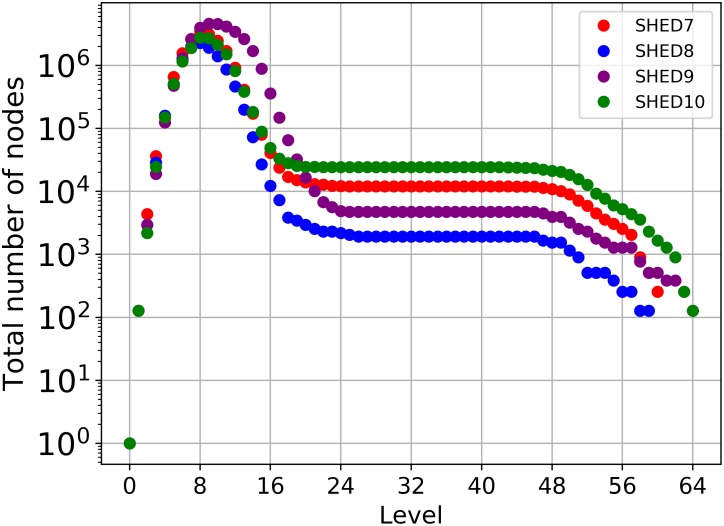
Total nodes per level.

Knowing at which level our 7-D Tree presented the greatest number of data points is important for the algorithm because this level indicated the break between the linear and asymptotic portion of the curves of the log-log plot of *log*(*N*(*ε*)) vs. *log*(1/*ε*). Fig 4 shows the total number of nodes containing data per level as a TreeMap. We only considered the levels between 3 and 14, as smaller or greater levels were marginal contributors in the top right corner of the diagram, and cluttered the presentation. Fig 4 shows that, regardless of dataset, the 8^th^ level, *ε* = 0.0005, contains the plurality of the data points. In addition, we can see in Fig 5 that the tree reached its maximum expansion around the 8^th^ level, which indicates that the tree starts to shrink after this level.

### Intrinsic dimensionality

**Fig 6:**
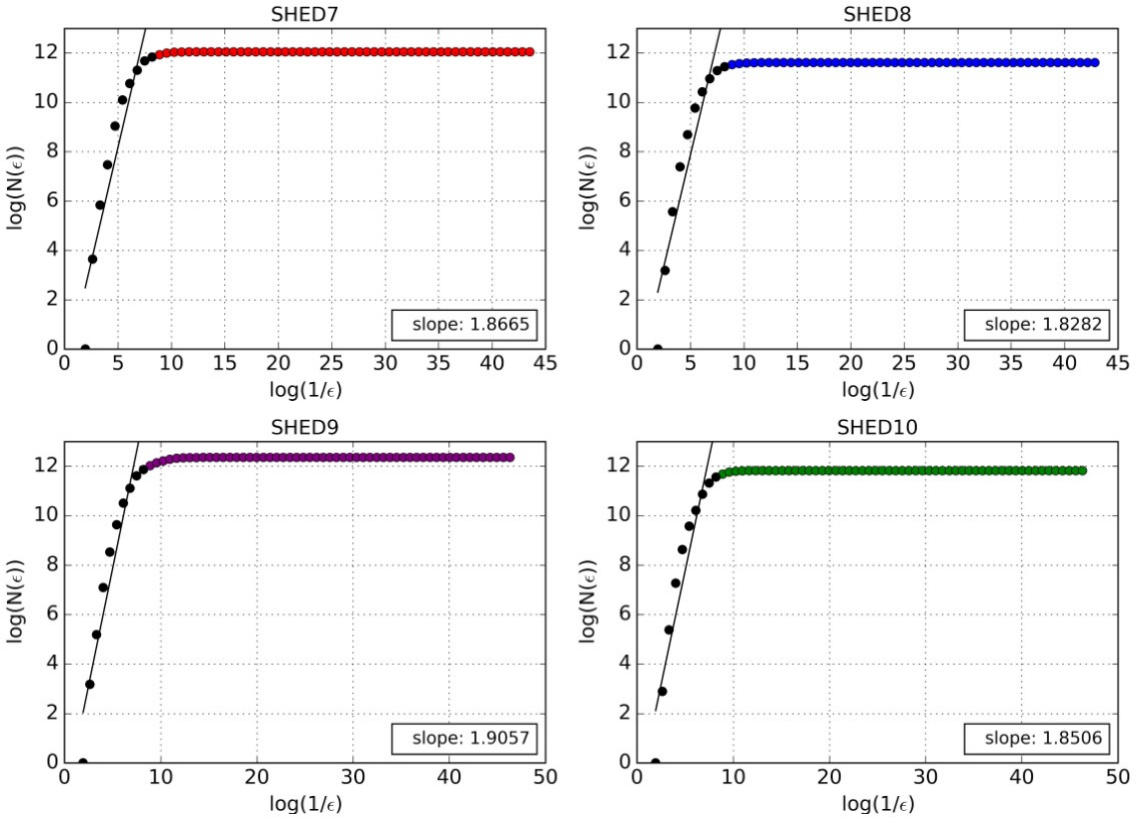
Slope calculation of the ID of each dataset. Black points represent the linear portion employed in slope calculation.

Fig 6 shows the best fitted lines for our *log*(*N*(*ε*)) vs. *log*(*ε*) plots. As described above, after the 8^th^ level, an asymptotic curve is formed. Since the asymptotic curve starts between the 8^th^ and 9^th^ levels, we considered the first 8^th^ and 9^th^ points as the linear part of our graph, and took the average between their slopes as the ID, which showed to be between 1.82 and 1.90. The ratio of the total points from the dataset inserted into the leaves until the 8^th^ and 9^th^ levels as well as the ID for each dataset can be seen in Table 4.

**Table 4:** Ratio of leaves and IDs.

Datasets	8th level	9th level	IDs
SHED7	54.43%	69.25%	1.86
SHED8	58.14%	74.72%	1.82
SHED9	32.13%	45.61%	1.90
SHED10	45%	64.69%	1.85

**Fig 7:**
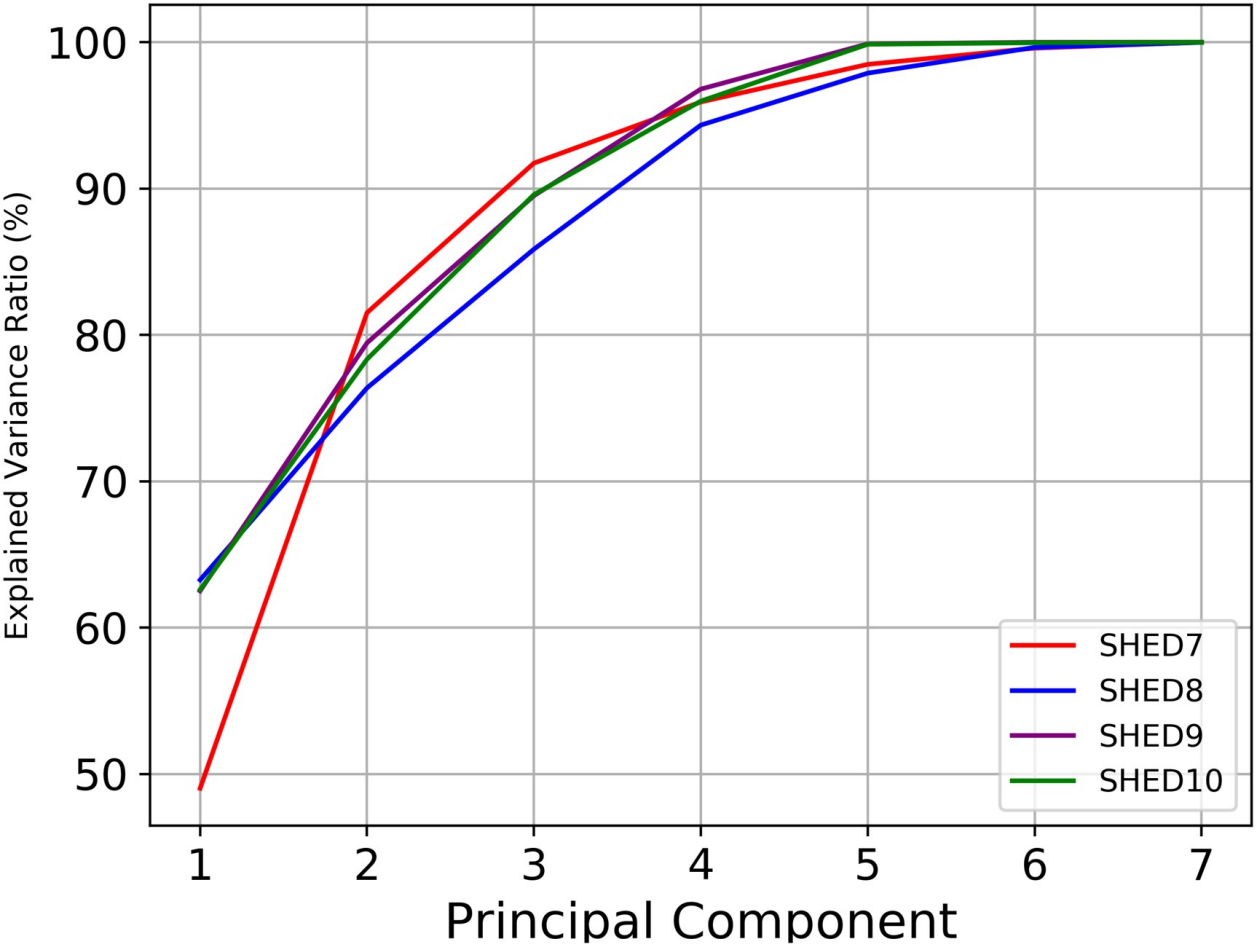
Principal component analysis. PCA result of the cumulative explained variance ratio. This figure indicates the amount of data that can be described from 1 to the maximum of principal components (number of dimensions of the dataset).

**Fig 8:**
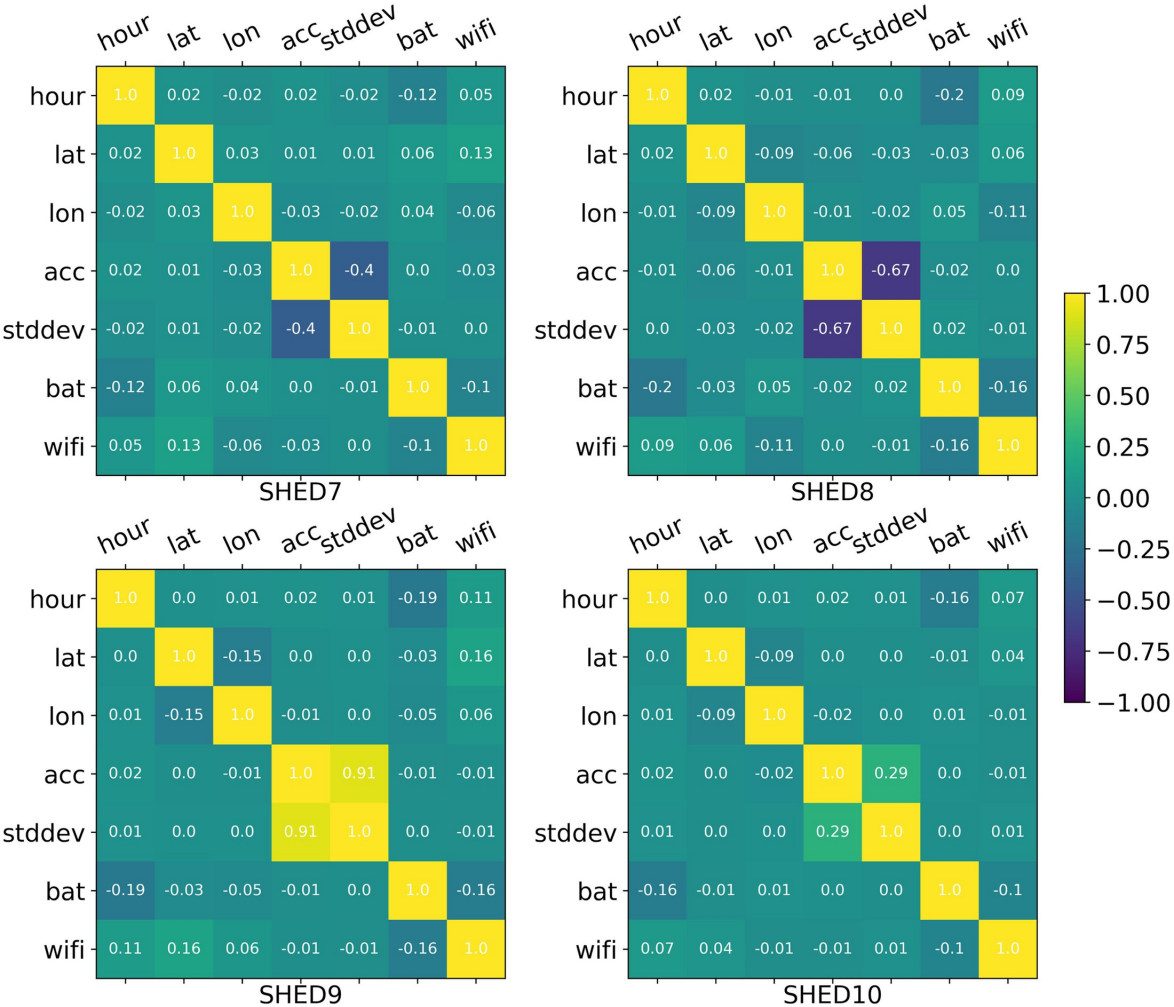
Correlation Matrix. Correlation analyses between all the 7 dimensions. Strong correlations are closer to 1 or -1 values.

We ran a PCA analysis using Python 3.6.1 with sklearn to determine the extent to which a linear correspondence could be found in the data. The PCA method showed that approximately 90% of the data from the SHEDs datasets can be described with 3 or 4 dimensions using a linear transformation, greater than the ID values resulted from Box Counting analysis. The PCA inertia is shown in Fig 7. This result clearly showed that the dimensions are not linearly correlated. This lack of linear correlation can be further confirmed using a Correlation Matrix. As expected, Fig 8 shows that only the accelerometer magnitude (*acc*) and its standard deviation (*stddev*) presented a strong linear correlation across all the datasets The majority of the correlations were close to 0 but, such as battery and hour had weak correlations. PCA did not provide consistency on its selection of the dimensions that better describe the datasets for the second and third principal component, PC2 and PC3, as shown in Table 5. For SHED7, the battery is the primary dimension for PC2 while for the other datasets, the longitude showed greater variance. For PC3, the opposite occurred. Due to the lack of correlation, and inconsistency of the eigenvectors, we conclude the ID of human behavioral activity is based on non-linear mappings between dimensions. These results do not invalidate the utility of the PCA or similar algorithms results which provide the new dimensions as well as an estimate of dimensionality, but instead serves to illustrate the difference between what the linear estimate returns and the intrinsic dimensionality of the data.

**Table 5:** Eigenvectors and eigenvalues for the first three principal components for each dataset.

PC1				
Dimens	SHED7	SHED8	SHED9	SHED10
hour	**-9.518e-01**	**-9.951e-01**	**-9.950e-01**	**-9.963e-01**
lat	-4.697e-04	-8.496e-03	-1.360e-03	1.464e-03
lon	1.354e-02	8.662e-03	-7.657e-03	-4.878e-03
wifi	-1.811e-02	-2.591e-02	-2.807e-02	-1.991e-02
bat	*3.057e-01*	*9.449e-02*	*9.450e-02*	*8.262e-02*
acc	-3.192e-03	6.417e-04	-6.354e-04	-8.427e-04
stddev	1.519e-03	2.109e-04	-4.522e-04	-2.834e-04
eigenvalue	0.097	0.094	0.091	0.090
PC2				
Dimens	SHED7	SHED8	SHED9	SHED10
hour	*3.062e-01*	-2.798e-02	1.599e-02	1.095e-03
lat	2.723e-02	*2.255e-01*	*1.646e-01*	*9.817e-02*
lon	2.477e-02	**-9.557e-01**	**-9.823e-01**	**-9.941e-01**
wifi	-2.626e-02	9.556e-02	-2.653e-02	1.038e-02
bat	**9.508e-01**	-1.606e-01	8.329e-02	-4.470e-02
acc	2.118e-03	-2.163e-04	7.845e-04	2.004e-03
stddev	-1.507e-03	4.703e-03	-1.231e-04	-2.287e-05
eigenvalue	0.064	0.019	0.024	0.022
PC3				
Dimens	SHED7	SHED8	SHED9	SHED10
hour	-5.396e-03	9.333e-02	9.539e-02	*8.402e-02*
lat	-2.996e-02	-1.354e-01	*-1.939e-01*	-1.321e-02
lon	**-9.984e-01**	*-2.075e-01*	5.459e-02	-4.664e-02
wifi	*3.592e-02*	-1.213e-01	-1.387e-01	-7.667e-02
bat	2.958e-02	**9.565e-01**	**9.649e-01**	**9.923e-01**
acc	8.667e-03	-5.480e-03	-4.923e-04	7.670e-04
stddev	3.851e-03	1.151e-02	3.386e-06	8.323e-05
eigenvalue	0.020	0.014	0.014	0.016

The most significant dimension is bold, the second is italic.

## Discussion

In this paper, we have demonstrated the utility of the Box Counting dimension for the characterization of diverse spatial datasets. Results indicated that, for the datasets considered, the fractal dimension of the dataset was remarkably consistent, with values between 1.8 and 1.9. A continuous dimension may appear a strange result, but it indeed describes the minimum dimension of the data. A famous example is the Sierpinsky triangle which has an ID between 1 and 2 because it is not an 1-D object due to its infinite perimeter nor a 2-D due to the lack of an area [35, 36]. In a human movement context, a person moving in a building would present an embedding dimension of 3: x, y, and z coordinates, where z can be discrete because it is attached to the floor. As a result, the full z dimension is not required. Fractal dimension attempts to quantify exactly how many dimensions are needed. As the data described here is spatial, one would initially expect a dimension of greater than two, representing longitude, latitude and the time of day. However, because both location and time are bounded and discrete (time explicitly by hour and space implicitly by the resolution of GPS), they can each be represented as finite arrays (time explicitly as a 24 bin array, and space as the total area divided by the resolution squared). Because all dimensions in the dataset can be regarded in this manner, as limited by intent or sensor resolution, all dimensions in the dataset are representable as single-dimensional entities, although the tables would be quite large for the continuous sensed phenomenon (position and activity). Because all measured dimensions (distinct from the continuous dimensions they represent) are finite and countable, they can be represented in the limit using less than a full dimension. However, we suspect that this does not describe the entirety of the dimensionality. Logical correlations between space-time and other human activities (for example one is unlikely to engage in calisthenics in the washroom), which would lead to mappings and constrains between dimensions reducing the dimension due to behavior. While some of the dimensionality can be attributed to the structure of the data, a significant portion must be attributed to the non-linear correspondences between features. The dimesionality is a reflection that human activity is inherently bounded (for example by the surface of the earth for most people), that sensors have a fundamental limit to their resolution, and that, as established by Song et al. [45], human spatial behavior has a high degree of predictability in the limit. What this dimensionality expresses is the extent of those intrinsic constraints for these particular datasets.

Analysis of the structure of the tree indicated that a maximum proportion of the data was contained in leaf at the 8th expansion, or 2^56^ discrete bins (the total number of hypercubes in this level if all the nodes were divided, (2^*d*^)^*l*^). After this level, the Box Counting algorithm did not locate a substantial number of new datapoints for an expansion. This indicates the minimum countable set that is required to reasonably represent the data, because aggregation to this level provides the most efficient and diverse representation of the data. While this approach does not describe the structure of the correlation, it can still be employed when evaluating features, as ID with and without a given feature can be calculated to establish the incremental impact of that feature [35].

A correlation analysis of the data indicated no obvious linear correlations, except between the mean and variance of the accelerometer norm, as expected. A PCA analysis similarly was inconclusive, with different datasets ranking different combinations of parameters as important in successive eigenvectors. The inconsistency of the PCA analysis across datasets was at odds with the consistency of the dimensionality. Box Counting by its nature is more accurate in determining the ID, as it does not make the linear assumptions of PCA. However, Box Counting only returns the ID in the limit, but provides no information on what those dimensions are. If the ID from Box Counting and PCA were the same, then the linear assumption would be valid, and the dimensions with most variances returned by PCA could be used with confidence. Because they were not the same with our datasets, the number of dimensions of PCA did not represent the smallest possible number. We would recommend that researchers employing PCA or similar techniques to provide a more concise representation of their data should also include an estimate of ID to establish the degree to which the representation approaches the theoretical optimum.

As a results of this analysis, we have made the following contributions to human behavioral activity data:

**Dimensional Analysis** We are the first, to the best of our knowledge, to propose and describe using the Box Counting dimension the structure of spatial behavior datasets that are now possible to collect from smartphones. This dimensionality is low considering the complexity of the variables involved, but sensible given the bounded, countable and predictable nature of sensed human behavior. These results additionally imply that much of human behavior is correlated, even if those correlations are not linear, or accessible to traditional statistical modeling.
**Tree Analysis** If the Box Counting dimension is generated using an n-D Tree decomposition, then the structure of the tree can be probed to generate insight into the structure of the data. This is a novel use of the Box Counting dimension and constitutes a methodological contribution in and of itself. The analysis of the datasets using the techniques showed that the maximum ratio of data-containing boxes occurred at 32 to 58 % of the total number of data points.
**Non-linearity** Both correlation and PCA were unable to consistently account for the low dimension across datasets, providing strong evidence that any correlations are non-linear. Furthermore, the fractal dimension obtained through Box Counting dimension is indicative of systems typified by feedback models. This result is sensible given the nature of the data studied, where future actions are strongly contingent on past states.

The technique we have described here is generic and could be employed for similar datasets. Code required for this analysis can be found at [46]. We would anticipate different results for intrinsic dimensionality and different tree structure for different input dimensions and participant demographics. The similarity of the dimensionality between datasets for the intrinsic dimension were striking, but these datasets were all built from similar demographics (students from a Canadian Prairie university). While we expect that the technique will generalize, and suspect that the intrinsic dimensionality of students at similar-sized universities in developed countries will likely be similar, we do not believe that the empirical results are universally true for all of human endeavour. The datasets we chose were of modest size. While Box Counting dimension itself scales relatively well, holding the tree in memory does not. Blindly using the code described here on large datasets could result in memory issues. This is trivially solved by not building the tree, or by using more eloquent coding techniques to manage tree size and expansion.

### Limitations and future work

While our paper constitutes an important contribution to the methodology of studying human spatial behavior, several limitations should be noted which point the way towards future inquiries. The four similar datasets studied demonstrate that the approach is technically sound, provides to be consistent and is computable in a reasonable amount of time for the scale of the datasets in question. However, the scope of human endeavour encompasses more than the habits of North American university students, and the results presented here must be regarded as a baseline. Further analysis of this method to determine its consistency across a wider variety of human (or non-human) populations to establish the variability of the measure, and larger datasets to establish the extent of computable solutions, constitute a potentially impactful body of future work. Because the IDs over the four similar datasets were consistent, an opportunity stemming from this work lies in studying dimensionality as a feature capable of meaningfully distinguishing between populations or behaviors. If the ID is constant for all demographics, that would be an interesting finding regarding human behavior. On the other hand, if the ID varies with demographics or behavior, then ID becomes a plausible feature for measure the human mobility complexity.

We treated datasets in their entirety and did not investigate the distribution of dimensionality at the participant level. This could be a fruitful avenue for future research, as dimensionality may be diagnostic of individual or population differences in the same manner as entropy rate [45]. We selected what we believed to be interesting and distinct features from the dataset to examine, but this set is illustrative rather than exhaustive or authoritative. Subsequent analyses across different or larger input vectors of human behavior could yield additional insight. Finally, this work ignores the likely impact of scale of analysis, often referred to in geography as the modifiable areal unit problem (MAUP) [47]. Because the dimensionality is related to the countability of the measurements, elements which change countability, like increases in spatio-temporal resolution, or the bounds of time and space considered could have an impact. The relationship between scale and dimension is a promising future research area.

## Conclusion

In this paper, we provided the first calculation of intrinsic dimensionality for human behavioral activity by analyzing seven smartphone sensor metrics over four datasets. We applied the Box Counting dimension to calculate the intrinsic dimensionality. By using a tree structure, the data was organized in a meaningful way that allowed us to compute the required parameters for the Box Counting dimension. Our methodology showed that the human behavioral activity can be described with a low dimension, between 1.8 and 1.9, while the linear dimensionality reduction technique PCA resulted between 3 and 4 dimensions to describe 90% of the data, indicating that the correspondence between dimensions was non-linear. Further work considering a dataset including diverse occupations and locations as well as analyzing the human activity by individual level can provide more insight of the intrinsic dimensionality of human behavior.
